# Insecticide resistance of *Anopheles sinensis* and *An. vagus* in Hainan Island, a malaria-endemic area of China

**DOI:** 10.1186/1756-3305-7-92

**Published:** 2014-03-03

**Authors:** Qian Qin, Yiji Li, Daibin Zhong, Ning Zhou, Xuelian Chang, Chunyuan Li, Liwang Cui, Guiyun Yan, Xiao-Guang Chen

**Affiliations:** 1 Key Laboratory of Prevention and Control for Emerging Infectious Diseases of Guangdong Higher Education Institutes, Department of Pathogen Biology, School of Public Health and Tropical Medicine, Southern Medical University, Guangzhou, Guangdong 510515, China; 2 Department of Parasitology, Wenzhou Medical University, Wenzhou 325035, China; 3 Program in Public Health, College of Health Sciences, University of California at Irvine, Irvine, CA 92697, USA; 4 Department of Entomology, Pennsylvania State University, State College, University Park, PA 16802, USA

**Keywords:** *Anopheles sinensis*, *Anopheles vagus*, Insecticide resistance, *Kdr* mutation, *Ace-1* mutation, Metabolic detoxification enzymes

## Abstract

**Background:**

Malaria is one of the most important public health problems in Southeast Asia, including Hainan Island, China. Vector control is the main malaria control measure, and insecticide resistance is a major concern for the effectiveness of chemical insecticide control programs. The objective of this study is to determine the resistance status of the main malaria vector species to pyrethroids and other insecticides recommended by the World Health Organization (WHO) for indoor residual sprays.

**Methods:**

The larvae and pupae of *Anopheles* mosquitoes were sampled from multiple sites in Hainan Island, and five sites yielded sufficient mosquitoes for insecticide susceptibility bioassays. Bioassays of female adult mosquitoes three days after emergence were conducted in the two most abundant species, *Anopheles sinensis* and *An. vagus,* using three insecticides (0.05% deltamethrin, 4% DDT, and 5% malathion) and following the WHO standard tube assay procedure. P450 monooxygenase, glutathione S-transferase and carboxylesterase activities were measured. Mutations at the knockdown resistance (*kdr*) gene and the *ace-1*gene were detected by DNA sequencing and PCR-RFLP analysis, respectively.

**Results:**

*An. sinensis* and *An. vagus* were the predominant *Anopheles* mosquito species. *An. sinensis* was found to be resistant to DDT and deltamethrin. *An. vagus* was susceptible to deltamethrin but resistant to DDT and malathion. Low *kdr* mutation (L1014F) frequency (<10%) was detected in *An. sinensis*, but no *kdr* mutation was detected in *An. vagus* populations. Modest to high (45%-75%) *ace-1* mutation frequency was found in *An. sinensis* populations, but no *ace-1* mutation was detected in *An. vagus* populations. Significantly higher P450 monooxygenase and carboxylesterase activities were detected in deltamethrin-resistant *An. sinensis*, and significantly higher P450 monooxygenase, glutathione S-transferase and carboxylesterase activities were found in malathion-resistant *An. vagus* mosquitoes.

**Conclusions:**

Multiple insecticide resistance was found in *An. sinensis* and *An. vagus* in Hainan Island, a malaria-endemic area of China. Cost-effective integrated vector control programs that go beyond synthetic insecticides are urgently needed.

## Background

Malaria is a major public health problem worldwide and has significantly impeded socioeconomic development. According to the latest World Health Organization (WHO) report, since 2000 the global malaria mortality rate has decreased by 45% [1]. In China, malaria incidence has been trending downward; the total number of malaria cases was reported to be less than 3,000 in 2012 [1]. The Chinese government issued “Action Plan of China Malaria Elimination (2010-2020),” aiming at malaria elimination in China by 2020 [2,3]. Pyrethroid-impregnated bed nets and indoor residual sprays are the main components of malaria control and elimination strategy because of the high mosquito repellency of pyrethroids, insecticidal efficacy, and low toxicity to mammals [4-7]. However, extensive use of insecticides has resulted in widespread pyrethroid resistance, and insecticide resistance in malaria vectors is a growing concern in many countries. Insecticide resistance monitoring and management is a high priority in malaria control programs.

Hainan Island Province is one of two provinces in China with endemic *Plasmodium falciparum* malaria in China [8]; here, *P. vivax* malaria is also endemic. Hainan Island, separated from mainland China by the 30 km-wide Qiongzhou Strait, has a size of 33,920 km^2^ and a population of 8.6 million. It has a tropical moist monsoonal climate with annual average temperature of 25°C and precipitation of 1500-2000 mm. The southern part of the island is mountainous and is the most malarious region. Recent malaria incidence reports showed that 11 out of 17 counties in the southern part of the island had malaria incidence exceeding 1/1000 [9,10]. Major malaria vectors in Hainan Island are *An. minimus* and *An. dirus* and the secondary malaria vector is *An. sinensis*[11-14]. Due to recent intensive malaria vector control measures, the abundance of *An. minimus* and *An. dirus* has been decreasing and *An. sinensis* becomes increasingly important, as found in the present and other studies [11,12,14]. Although so far *An. vagus* has not been confirmed as a malaria vector in Hainan, *An. vagus* has been reported as a malaria vector in Vietnam, Laos, Cambodia [15,16], Central Java (Purworejo, Kokap) and western Timor Island (Kupang) in Indonesia (*P. falciparum*) [17] and Bangladesh (*P. falciparum* and *P. vivax*) [18]. Chemical insecticides have been extensively used since the 1950s to control mosquito vectors, and historically, four major categories of insecticides have been sequentially applied: organochlorines, organophosphates, carbamates, and pyrethroids [19]. Pyrethroids are the most commonly used insecticides for insecticide-treated nets (ITN) and indoor residual spraying (IRS), which target indoor transmission and indoor-biting and–resting mosquitoes. Extensive use of pyrethroid insecticides has resulted in insecticide resistance, which has changed the mosquito vector community and population structures in Hainan Province. For example, *An. sinensis*, an exophagic and exophilic species, has now become a very important malaria vector due to its wide distribution, high density, and prevalent insecticide resistance [11,19,20].

Two insecticide resistance mechanisms have been identified in mosquitoes: increased metabolic detoxification and reduced target site sensitivity [21]. Metabolic detoxification enzymes include cytochrome P450 monooxygenases (P450s), glutathione S-transferases (GSTs) and carboxylesterases (COEs). Pyrethroids and organochlorines function as neurotoxins that act by prolonging sodium channel activation, whereas organophosphates and carbamates kill insects by inhibiting acetylcholinesterase found in the central nervous system [22-24]. Target site resistance to pyrethroids and organochlorines is caused by mutations in the *para*-type sodium channel gene, while target site resistance to organophosphates and carbamates is caused by a mutation at codon 119 of the acetylcholinesterase (*ace-1*) gene. Mutations at the *para*-type sodium channel gene cause knockdown resistance (*kdr*), and *kdr* frequency has been used as an index of mosquito resistance to pyrethroids [25-27]. In *An. sinensis*, four non-synonymous mutations at codon 1014 were reported, including L1014F [28-30], L1014C [28-30], L1014S [16,31], and L1014W [29]. In *An. gambiae*, the most important malaria vector in Africa, a mutation at codon N1575Y augments pyrethroid resistance [32]. On the other hand, a mutation at codon 119 of the *ace-1* gene leading to a single amino acid substitution of glycine to serine in the binding pocket of acetylcholinesterase may confer resistance to organophosphates and carbamates. Mosquitoes have two acetylcholinesterase genes (*ace-1* and *ace-2*), but only *ace-1* was found to be significantly associated with insecticide resistance [33-35].

The objective of the present study was to determine the spatial heterogeneity of pyrethroid resistance in malaria vectors in Hainan Island, China. Because organochlorines and organophosphates were used for vector control and have been considered for IRS, we also determined resistance to these two classes of insecticides. We examined the frequency of *kdr* and *ace-1* mutations and analyzed metabolic detoxification enzyme activities to determine the spatial distribution of *kdr* and *ace-1* mutations and to ascertain whether any molecular or biochemical biomarkers may be predictive of insecticide resistance.

## Methods

### Mosquito sample collection

**Figure 1:**
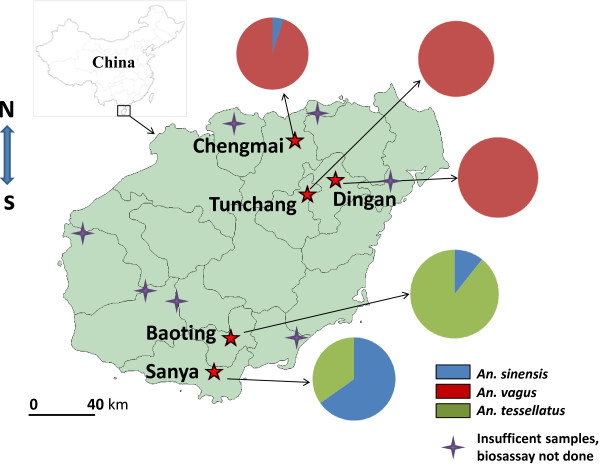
**A map of *Anopheles *mosquito sampling sites in Hainan Island, China.** Pie chart shows the species composition.

In order to minimize the confounding effects of age and blood feeding history on insecticide resistance in field-collected adult mosquitoes [36], we used female adult mosquitoes reared from field-collected larvae or pupae for the resistance bioassay. *Anopheles* mosquito larvae and pupae were collected from 12 localities in Hainan Island Province, China, in July–August 2012 (Figure 1). In each locality, at least 80 aquatic habitats in rice fields were sampled using 350 ml larval dippers. Malaria is endemic in all sites, but the southern part of the island had higher malaria incidence. Insecticides have been used extensively for agricultural pest and mosquito control. The main insecticides used in these areas include pyrethroids in mosquito coils, DDT, lambda-cyhalothrin, methothrin, permethrin, triazophos and triazophosphoxim. In each site, we collected as many mosquito larvae and pupae as possible from the rice fields. Mosquito larvae and pupae were reared to adults under local environmental conditions. Prior to insecticide assay, all adult mosquitoes were identified to species morphologically using the published morphological keys of Done 2010 [37]. Seven of the 12 sites yielded <100 *Anopheles* mosquitoes; therefore, insecticide resistance bioassay was not conducted due to small sample size.

### Mosquito insecticide susceptibility bioassay

We used the standard WHO insecticide susceptibility tube test [27] to determine susceptibility to deltamethrin, DDT, and malathion in *An. sinensis* and *An. vagus* wherever a sufficiently large number of mosquitoes was collected*.* Test papers (supplied by China CDC) were 0.05% deltamethrin, 4.0% DDT, and 5% malathion. In order to minimize the effect of mosquito age and blood feeding history on resistance, we used female adult mosquitoes three days after emergence without blood-feeding for all experiments. The susceptible control population was a laboratory *An. sinensis* strain that has been maintained in the insectary of the Jiangsu Institute of Parasitic Diseases in Wuxi, China, for over 10 years [30]. For each insecticide, a total of 100–200 female mosquitoes were tested with 20-25 mosquitoes per tube. Silicone oil-treated papers without insecticide (control paper) were included in the test. After a 1 hr exposure, mosquitoes were transferred to recovery cups and maintained on 10% sucrose solution for 24 hrs. Mosquito survivorship was recorded hourly during the 24 hr recovery period. Here we defined “resistant” as the mosquitoes that were alive 24 hours after 60-min exposure to the insecticides in the standard WHO tube bioassay, and “susceptible” as the mosquitoes that were knocked down within the 24-hr recovery period [38]. Mosquitoes were considered knocked down if they were unable to walk from the center to the border of a 7-cm filter paper disc, either alone or when they were mechanically stimulated [39]. After the resistance/susceptible status was recorded, one leg of each mosquito was removed and preserved individually in 95% alcohol for subsequent DNA analysis, and the remaining mosquito body was immediately tested for metabolic enzyme activities. Therefore, only fresh mosquitoes were tested for metabolic enzyme activities.

### Metabolic enzyme activity assays

Three metabolic enzymes were analyzed in individual mosquitoes: cytochrome P450 monooxygenases (P450s), glutathione S-transferases (GSTs), and carboxylesterases (COEs). Measurement of P450 and GST activity followed the methods of Penilla *et al.*[40] and Zhong *et al.*[30]. COE activity was measured following the method of Hosokawa and Satoh [41]. Total protein was measured for each mosquito using the method of Bradford [42]. Mean absorbance values for each tested mosquito and enzyme were converted into enzyme activity and standardized based on the total protein amount. P450 and GST activities were calculated as pmol 7-HC/min/mg protein and μmol cDNB/min/mg protein, respectively. COE activity was calculated as μmol p-nitrophenol/min/mg protein, using the formula (Δabsorbance/min–Δblank/min) × 1.0/16.4 × 0.05 × protein (mg/ml). An absorption coefficient of 16,400 M^-1^ · cm^-1^ was used [43]. All measurements were done in duplicate. For each mosquito population and each insecticide, 100 adult female mosquitoes were tested. Although exposure to insecticides may alter the metabolic enzyme level, the ratio of metabolic enzyme level in the resistant mosquitoes to the susceptible mosquitoes should reflect the relative difference between resistant and susceptible individuals because the resistant and susceptible mosquitoes were both exposed to the insecticides in the same manner.

### Mosquito DNA extraction and molecular identification of mosquito species

One leg of a single mosquito was used for DNA extraction with the EZNA™ Micro Elute Genomic DNA Kit (Promega, Madison, WI). Molecular identifications of *An. sinensis* and *An. vagus* species were conducted using species-specific PCR primers (forward: TGTGAACTGCAGGACACATGAA and reverse: AGGGTCAAGGCATACAGAAGGC for *An. sinensis*; forward: CACACATCCTTGAGTGCTA and reverse: ACACATCACTTGAGGCCAC for *An. vagus*) to amplify the second internal transcribed spacer (ITS2) and 28S-D3 rDNA regions [44,45]. Amplification was performed in a 25 μL reaction containing 2 μL of template DNA, 2.5 μL of 10 × PCR buffer, 0.75 μL of 50 mM MgCl_2_, 2 μL of 2.5 mM of each dNTP, 0.5 μL of 10 μM of each primer, and 0.625unit Platinum®Taq DNA Polymerase (TaKaRa, China). The cycling conditions were as follows: initial denaturation at 94°C for 2 min, 30 cycles of 30s denaturation at 94°C, 30s annealing at 48°C and 30s extension at 72°C followed by a final extension of 10 min at 72°C. Amplification products were examined on a 2.5% agarose gel electrophoresis. The species was determined by the size of the PCR product (1,077 bp for *An. sinensis* and 604 bp for *An. vagus*). Molecular identification was conducted for all mosquitoes subjected to insecticide resistance bioassay.

### Detection of *kdr* mutation and *ace-1* mutation

To determine point mutations of the *kdr* gene at codon 1014, we amplified a 325 bp fragment in *An. sinensis* and a 258 bp fragment in *An. vagus* flanking the codon 1014, following the methods previously described by Zhong *et al.*[30] and Verhaeghen *et al.*[16]. The PCR product was directly sequenced from both ends using the same PCR primers by the Life Genetic Service Facility (Invitrogen, Shanghai, China). To detect point mutations of the *ace-1* gene at codon 119 in *An. sinensis* and *An. vagus*, a PCR-RFLP method was used [35,46,47]. Briefly, we designed a pair of primers (forward: GTGCGACCATGTGGAACC and reverse: ACCACGATCACGTTCTCCTC) based on the *An. gambiae ace-1* gene sequence (GenBank accession: BN000066) to amplify a 193 bp fragment that flanks the target codon position 119 in the *ace-1* gene. The PCR product was digested by *Alu*I restriction enzyme, which results in 118 bp and 75 bp fragments when there is a homozygous G119S mutation. Homozygous wildtype results in no restriction digestion (i.e., the result is a 193 bp fragment). A total of 267 *An. sinensis* mosquitoes and 300 *An. vagus* mosquitoes were sequenced for *kdr* and genotyped for *ace-1* mutations by PCR-RFLP.

### Statistical analysis

Mosquito mortality rates after the 24 hr recovery period were calculated for each insecticide and each population. The corrected mortality rates using the Abbott’s formula [48] were reported. We classified mosquito resistance status according to WHO criteria [27]: resistant if mortality is <90%, probably resistant if mortality is 90%-98%, and susceptible if mortality is >98%. Univariate analysis of variance (ANOVA) was conducted using the *arcsin* transformation of the mosquito mortality rate to determine among-population differences in mosquito mortality rates in the insecticide susceptibility bioassay. One-tailed Mann-Whitney U tests were used to compare the enzyme activities between resistant and susceptible mosquitoes for each population. Chi-square tests were used to examine the association between target site mutations and the resistance phenotype.

## Results

### *Anopheles* mosquito species composition in Hainan Island

A total of 10,975 *Anopheles* mosquito larvae and pupae were collected in the 12 sites; four sites (Ledong, Wuzhishan, Dongfang and Haikou) did not yield any collection. Among the collected mosquitoes, three species were identified based on morphological characteristics. *An. vagus* was the predominant species in three sites: Tunchang (100%), Dingan (100%) and Chengmai (95%) (Figure 1). *An. sinensis* was predominant in Sanya (65.2%) and *An. tessellates* in Baoting (89.3%). *An. vagus* and *An. tessellates* were also collected in three other sites (Lingao, Linshui and Wenchang), but fewer than 100 specimens were collected, and thus vector species composition was not calculated.

### Insecticide susceptibility bioassay and molecular identification of species

Insecticide resistance bioassay was conducted in five mosquito populations, including *An. sinensis* populations from Sanya and Baoting and *An. vagus* from Tunchang, Dingan and Chengmai. Mortality rates of *An. sinensis* mosquitoes from Sanya and Baoting ranged from 85.8% to 91.0% when tested against deltamethrin and from 72.7% to 78.4% against DDT, suggesting that *An. sinensis* was resistant to DDT and resistant or probably resistant to deltamethrin, based on the WHO criteria. Mortality rates of *An. vagus* mosquitoes were high (97.9%–100%) against deltamethrin but <90% against DDT and malathion for the three populations tested (Chengmai, Dingan and Tunchang) (Table 1). Therefore, *An. vagus* mosquitoes were resistant to DDT and malathion but susceptible to deltamethrin. In general, *An. sinensis* populations were more resistant than *An. vagus* to deltamethrin and DDT.

**Table 1:** **Mortality rate of insecticide resistance bioassay in *Anopheles sinensis *** **and *Anopheles vagus *** **mosquitoes from Hainan Island, China**

**Insecticide**	**Species**	**N**	**Site**	**Mortality (%) ± standard error**
**0.05% Deltamethrin**	*Anopheles sinensis*	152	Sanya	85.8 ± 6.2
		165	Baoting	91.0 ± 3.3
	*Anopheles vagus*	157	Chengmai	97.9 ± 1.5
		104	Dingan	100.0
**4% DDT**	*Anopheles sinensis*	102	Sanya	78.4 ± 7.1
		112	Baoting	72.7 ± 8.6
	*Anopheles vagus*	186	Chengmai	84.0 ± 3.7
		101	Dingan	88.8 ± 3.2
		88	Tunchang	67.1 ± 3.3
**5% Malathion**	*Anopheles vagus*	108	Chengmai	88.9 ± 1.9
		107	Dingan	77.3 ± 4.7
		86	Tunchang	78.9 ± 4.7

### Molecular species identification and *kdr* and *ace-1* allele frequencies

We confirmed our morphological species identification by performing rDNA PCR on 267 *An. sinensis* and 300 *An. vagus* mosquitoes. All mosquitoes were identified by their morphology and confirmed by molecular methods. The two methods gave consistent results. These same mosquitoes were sequenced to detect *kdr* mutation at codon 1014 of the *para*-type sodium channel gene. Only one type of *kdr* mutation (TTG to TTT) was detected at position 1014 in *An. sinensis* populations; this mutation leads to a change from leucine to phenylalanine (L1014F). Ten haplotypes were identified by DNA sequencing for *An. sinensis* (GenBank accession numbers: KF718269–KF718278). No homozygous *kdr* mutation genotype was detected. Heterozygous *kdr* genotype was detected in resistant mosquitoes but not in susceptible mosquitoes. The frequencies of *kdr* mutation in resistant *An. sinensis* mosquitoes were low, ranging from 6.7% (Baoting) to 9.5% (Sanya) (Table 2). Significant association was detected between *kdr* mutation and deltamethrin or DDT resistant phenotypes (P < 0.05) (Table 2). No *kdr* mutation was detected in either the *An. vagus* populations or the laboratory susceptible strain.

**Table 2:** ***Kdr *** **and *ace-1*** **genotype and mutation frequency in *Anopheles sinensis *** **mosquitoes from Hainan Island, China**

**Insecticide**	**Population**	Bioassay*	**N**	***Kdr*** **genotype**		**L1014F frequency**	**P**	***Ace-1*** **genotype**			**G119S frequency**	**P**
				**TTG/TTG**	**TTG/TTT**	**(%)**		**GGC/GGC**	**GGC/AGC**	**AGC/AGC**	**(%)**	
**0.05% Deltamethrin**	Sanya	Resistant	20	17	3	**7.5**	**0.041**	0	11	9	**72.5**	1.000
		Susceptible	47	47	0	**0**		1	26	20	**70.2**	
	Baoting	Resistant	15	13	2	**6.7**	0.090	2	7	6	**63.3**	0.435
		Susceptible	48	48	0	**0**		10	21	17	**57.3**	
**4% DDT**	Sanya	Resistant	21	17	4	**9.5**	**0.027**	0	13	8	**69.0**	1.000
		Susceptible	38	38	0	**0**		1	20	17	**71.1**	
	Baoting	Resistant	32	27	5	**7.8**	**0.024**	7	9	16	**64.1**	0.238
		Susceptible	46	46	0	**0**		13	17	16	**53.3**	

*“Resistant” refers to the mosquitoes that were alive 24 hours after 60-min exposure to the insecticides in the standard WHO tube bioassay; and “susceptible” refers to the mosquitoes that were knocked down within the 24-hr recovery period. Significant p-values (p < 0.05) are given in bold type.

The same *An. sinensis* and *An. vagus* mosquitoes analyzed for *kdr* mutations were genotyped for *ace-1*mutation at codon position 119 (G119S) by PCR-RFLP. DNA sequencing analysis identified 4 haplotypes in *An. sinensis* and 1 haplotype in *An. vagus* populations (GenBank accession numbers: KF718282–KF718286). The *ace-1* mutation was detected in the two *An. sinensis* populations (Sanya and Baoting) with high frequency, ranging from 63.3% to 72.5% in resistant mosquitoes and 53.3% to 71.1% in susceptible mosquitoes. No significant association was detected between *ace-1* mutation and deltamethrin and DDT resistant phenotypes (P > 0.05) (Table 2). The *ace-1* mutation was not detected in the three *An. vagus* populations or in the laboratory susceptible strain.

### Metabolic enzyme activity assays and association with resistance

**Figure 2:**
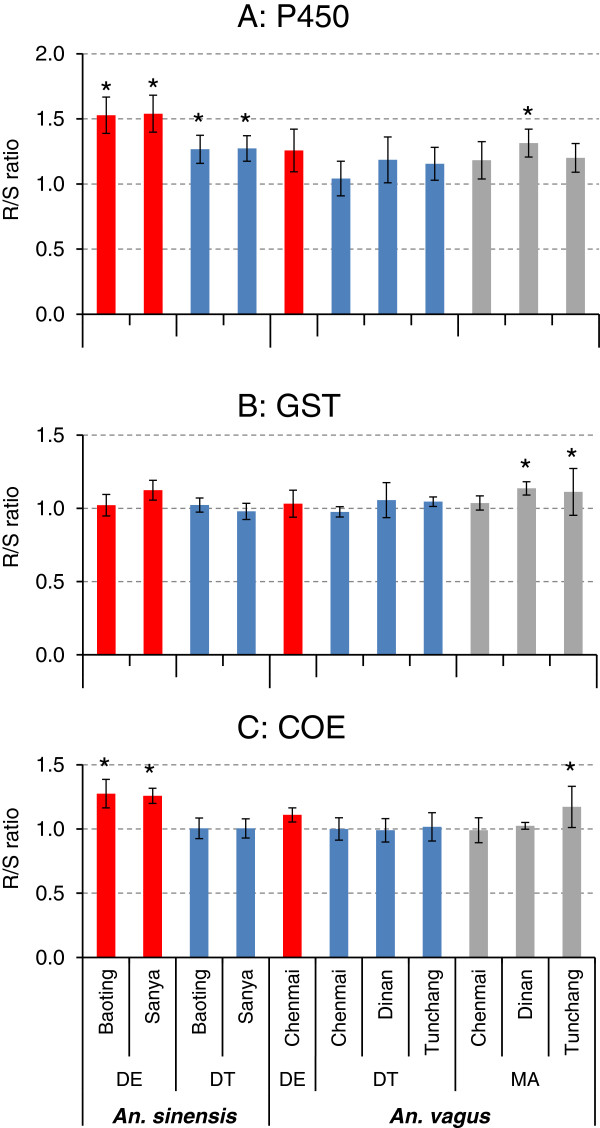
**Ratio of metabolic enzyme activity of resistant mosquitoes to that of susceptible mosquitoes (DE:Deltamethrin; DT:DDT; MA:malathion).** Top panel **(A)**: P450 monooxygenases (P450); middle panel **(B)**: glutathione s-transferase (GST); bottom panel **(C)**: carboxylesterases (COE).“*” indicates the ratio was significantly higher than 1.0 at p < 0.05.

The metabolic enzyme activity ratio of resistant mosquitoes (R) to susceptible mosquitoes (S), or R/S ratio, varied between species and among insecticides tested (Figure 2). The P450 enzyme activities were significantly higher in deltamethrin- and DDT-resistant mosquitoes in the two *An. sinensis* populations tested, as evidenced by R/S ratios significantly >1.0. Similarly, deltamethrin-resistant mosquitoes showed significantly higher COE enzyme activities in *An. sinensis*. No significant change in P450, GST and COE activities was detected in the three *An. vagus* populations when tested against deltamethrin and DDT. The P450, GST and COE enzyme activities were significantly higher in two malathion-resistant *An. vagus* populations.

## Discussion

Hainan Island is geographically isolated from the mainland of China and has been the most malarious region in China. Synthetic insecticide-based vector control is the primary malaria prevention and control method, thus determination of insecticide resistance status is paramount to the deployment of appropriate insecticides for malaria vector control. The present study surveyed the insecticide resistance status and determined the *kdr* and *ace-1* mutation frequencies in *An. sinensis* and *An. vagus* in Hainan Island. We found that *An. sinensis* populations have developed resistance to the three classes of insecticides tested, including pyrethroid (deltamethrin), organochlorine (DDT) and organophosphate (malathion). Multiple insecticide resistance in *An. sinensis* suggests that the insecticide currently used for bednet impregnation and IRS may not be able to provide effective protection to humans from parasite-carrying mosquitoes. Further examination of the resistance mechanism indicated that the mutation frequency of the *kdr* target site (L1014F) was significantly associated with deltamethrin and DDT resistance, and P450 and COE enzyme activities may also play an important role in *An. sinensis. An. vagus* was susceptible to deltamethrin but resistant to DDT and malathion. Interestingly, no *kdr* or *ace-1* mutations were detected in the 300 *An. vagus* specimens we analyzed. Elevated P450, GST and COE enzyme activities were found in malathion-resistant *An. vagus*, suggesting that metabolic resistance is the major resistance mechanism. However, it is also possible that this mosquito species exhibits behavioral avoidance to insecticides. Behavioral responses of mosquitoes to DDT and pyrethroids have been previously reported in *An. vagus*[49].

There have been debates in the literature over whether *kdr* frequency may be used as a biomarker for deltamethrin resistance [50,51]. In the two *An. sinensis* populations examined in the present study, we found that *kdr* frequency was significantly associated with the deltamethrin-resistant phenotype, and no L1014F allele was found in the susceptible individuals. Whether *kdr* frequency can predict deltamethrin resistance at the population level may be contingent on a number of factors, such as *kdr* frequency and the relative importance of metabolic resistance. The two *An. sinensis* populations examined here exhibited a low *kdr* mutation frequency (<10%), suggesting prevalent mutations in the *kdr* target gene. In central Chinese provinces such as Hunan, Hubei, Jiangsu and Anhui, *kdr* frequency in* An. sinensis* was very high (>90%) and *kdr* allele frequency had no predictive power of deltamethrin resistance [29,30]. On the other hand, *kdr* allele frequency had no predictive power when the populations completely lacked *kdr* mutations, as in the *An. sinensis* populations in Yunnan province [30]. Similar phenomena were found in *An. gambiae* and *An. arabiensis* mosquitoes in Africa, where *kdr* frequency was not significantly associated with pyrethroid resistance when *kdr* frequency was high [52,53], or when *kdr* mutation was completely lacking in the population [54].

Four types of *kdr* mutations (L1014F, L1014C, L1014S, and L1014W) have been reported in *An. sinensis* mosquitoes [16,28-31] and two types (L1014F and L1014S) in *An. vagus*[16,55]. Low frequencies of L1014F *kdr* allele in the heterozygous state were found in *An. sinensis* mosquito populations examined in our study. Such a low diversity of *kdr* mutations in Hainan Island mosquito populations may result from the geographic isolation of the island, which may prevent *kdr* mutations from being introduced to Hainan. Further study is needed to examine the genetic structure of *An. sinensis* populations in mainland China and Hainan Island to determine the role of gene flow in the spread of *kdr* mutations.

We did not detect any *kdr* or *ace-1* mutationsin *An. vagus* populations, which suggests either that *An. vagus* lacks mutations at the target sites of insecticides or that the mutation frequency was extremely low. The lack of mutations at the target sites of insecticides in *An. vagus* was not unique to our particular study sites. For example, a survey of *An. vagus* from 42 sites in Vietnam, Laos, and Cambodia did not detect *kdr* mutations in 35 sites, and the remaining 7 sites exhibited very low *kdr* frequencies [16]. Given the absence of mutations at the insecticide target sites, metabolic resistance becomes the major resistance mechanism. In the present study, we found significantly higher P450, GST and COE enzyme activities in malathion-resistant *An. vagus* individuals and significantly higher P450 and COE enzyme activities in deltamethrin-resistant *An. sinensis.* These results suggest that metabolic detoxification mechanisms were widespread in *An. sinensis* and *An. vagus* populations from Hainan Island. We want to note that all mosquito samples used for detoxification enzyme activity were pre-exposed to insecticide. It is possible that resistant and susceptible mosquitoes may exhibit differential responses to pre-exposure to insecticides in their detoxification enzyme activities. Therefore, our experimental procedure may potentially lead to skewing of R/S ratio.

Resistance to multiple classes of insecticides is becoming a common problem in various malaria vector species. Reported multiple resistance in malaria vectors includes *An. gambiae*[46,56-58], *An. arabiensis*[47], and *An. funestus*[59] in Africa, and *An. culicifacies, An. subpictus, An. nigerrimus,* and *An. peditaeniatus*[60] in Asia. Multiple insecticide resistance impedes the effectiveness of front-line malaria vector control programs, which are primarily based on the use of pyrethroids. These control programs have led to major changes in vectorial systems, including vector species composition as well as early biting behaviors and increased insecticide resistance in the past decade [61,62]; consequently, outdoor transmission has increased [63]. Considering the prevailing resistance to pyrethroids and organophosphates, the choice for synthetic insecticides is limited. Integrated vector control programs that go beyond synthetic insecticides but remain cost effective are urgently needed. Promising methods include larval source reduction through ecological or environmental manipulation [64,65], house modification [66], biopesticides [67], and long-lasting microbial insecticides [68]. Vector control tools with a long-lasting efficacy would help maintain the cost-effectiveness of the control program.

## Conclusion

This study found multiple insecticide resistance in *An. sinensis* and *An. vagus* in Hainan Island, a malaria-endemic area of China. Low *kdr* mutation frequency and modest *ace-1* mutation frequency were found in *An. sinensis. Kdr* frequency was significantly associated with deltamethrin resistance in *An. sinensis*, and significantly higher metabolic enzyme activities were found in resistant mosquitoes, suggesting that target-site insensitivity and metabolic resistance both play important roles in insecticide resistance in *An. sinensis*. In *An. vagus*, the absence of *kdr* and *ace-1* mutations and significantly higher P450, GST, and COE enzyme activities in malathion-resistant mosquitoes suggest that metabolic resistance is the major resistance mechanism. Cost-effective integrated vector control programs that go beyond synthetic insecticides are urgently needed.

## Competing interests

The authors declare that they have no competing interests.

## Authors’ contributions

All the authors have contributed significantly to this study. Conceived and designed the experiments: XGC and LC. Performed the experiments: QQ, YL, DZ, NZ and CL. Analyzed the data: QQ, DZ and GY. Wrote and revised the manuscript: QQ, DZ, GY, XC. All authors read and approved the final manuscript.
